# Supplementary material for: Circulating proteomic patterns in AF related left atrial remodeling indicate involvement of coagulation and complement cascade
Source: PLoS One. 2018 Nov 29;13(11):e0198461. doi: 10.1371/journal.pone.0198461 (PMC6264811; doi:10.1371/journal.pone.0198461)
Supplement: S1 Table — UP–number of unique peptides belonging to the protein which were detected in the screening, p-value—from T-Test comparing LVA and controls, p-value (FDR)–Benjamini Hochberg false discovery rate, FC–fold change in protein levels. Proteins in bold were found significantly different in LVA group compared to controls. (DOC) [file pone.0198461.s001.doc]

**Supplementary data 1**

**Table**: Proteins detected in proteomic approach comparing 23 patients with LVA and 25 patients without LVA. UP – number of unique peptides belonging to the protein which were detected in the screening, p-value - from T-Test comparing LVA and controls, p-value (FDR) – Benjamini Hochberg false discovery rate, FC – fold change in protein levels. Proteins in bold were found significantly different in LVA group compared to controls.

| **Protein names** | **UP** | **p-value** | **p-value (FDR)** | **FC** | **UniProt Entry** | **alternative protein names** | **gene symbol** |
| --- | --- | --- | --- | --- | --- | --- | --- |
| 14-3-3 protein zeta/delta | 1 | 0.604 | 0.762 | 1.25 | P63104 | KCIP-1 | YWHAZ |
| 72 kDa type IV collagenase | 2 | 0.013 | 0.081 | -4.56 | P08253 | TBE-1 | MMP2 |
| 78 kDa glucose-regulated protein | 1 | NA | NA | NA | P11021 | BiP | HSPA5 |
| Actin aortic smooth muscle | 1 | 0.334 | 0.599 | -1.20 | P62736 | Cell growth-inhibiting gene 46 protein | ACTA2 |
| Actin cytoplasmic 1 | 4 | 0.302 | 0.572 | -1.21 | P60709 | Beta-actin | ACTB |
| Adenylyl cyclase-associated protein 1 | 1 | NA | NA | NA | Q01518 | CAP 1 | CAP1 |
| Adiponectin | 3 | 0.608 | 0.762 | -1.10 | Q15848 | Gelatin-binding protein | ADIPOQ |
| Afamin | 13 | 0.224 | 0.483 | -1.09 | P43652 | Alpha-Alb | AFM |
| Alcohol dehydrogenase 1 | 8 | 0.084 | 0.270 | 1.14 | P00330 | YADH-1 | ADH1 |
| Alpha-1-acid glycoprotein 1 | 6 | 0.253 | 0.517 | 1.11 | P02763 | OMD 1 | ORM1 |
| Alpha-1-acid glycoprotein 2 | 5 | 0.099 | 0.293 | -1.14 | P19652 | OMD 2 | ORM2 |
| Alpha-1-antichymotrypsin | 16 | 0.188 | 0.436 | 1.12 | P01011 | Serpin A3 | SERPINA3 |
| Alpha-1-antitrypsin | 8 | 0.575 | 0.753 | -1.24 | P01009 | SPAAT | SERPINA1 |
| Alpha-1B-glycoprotein | 12 | 0.585 | 0.753 | -1.04 | P04217 | Alpha-1-B glycoprotein | A1BG |
| Alpha-2-antiplasmin | 10 | 0.356 | 0.623 | -1.05 | P08697 | Serpin F2 | SERPINF2 |
| Alpha-2-HS-glycoprotein | 13 | 0.629 | 0.785 | -1.03 | P02765 | Fetuin-A | AHSG |
| Alpha-2-macroglobulin | 56 | 0.422 | 0.659 | -1.08 | P01023 | C3 and PZP-like alpha-2-macroglobulin domain-containing protein 5 | A2M |
| Alpha-actinin-1 | 3 | 0.440 | 0.673 | 1.44 | P12814 | Non-muscle alpha-actinin-1 | ACTN1 |
| Aminopeptidase N | 1 | 0.681 | 0.827 | 1.19 | P15144 | CD antigen CD13 | ANPEP |
| Angiotensinogen | 11 | 0.929 | 0.937 | 1.01 | P01019 | Ang IV; Angiotensin 1-9; Angiotensin 1-7; Angiotensin 1-5; Angiotensin 1-4 | AGT |
| Antithrombin-III | 16 | 0.897 | 0.919 | 1.01 | P01008 | Serpin C1 | SERPINC1 |
| Apolipoprotein | 10 | 0.761 | 0.847 | 1.14 | P08519 | EC 3.4.21.- | LPA |
| Apolipoprotein A-I | 18 | 0.063 | 0.220 | -1.13 | P02647 | Apolipoprotein A-I | APOA1 |
| Apolipoprotein A-II | 4 | 0.579 | 0.753 | -1.05 | P02652 | Apolipoprotein A-II | APOA2 |
| Apolipoprotein A-IV | 18 | 0.906 | 0.919 | 1.02 | P06727 | Apolipoprotein A4 | APOA4 |
| Apolipoprotein B-100 | 172 | 0.503 | 0.702 | -1.09 | P04114 | Apo B-48 | APOB |
| Apolipoprotein C-I | 2 | 0.889 | 0.919 | 1.02 | P02654 | Apolipoprotein C1 | APOC1 |
| Apolipoprotein C-II | 3 | 0.437 | 0.673 | -1.15 | P02655 | ProapoC-II | APOC2 |
| Apolipoprotein C-III | 2 | 0.363 | 0.623 | -1.10 | P02656 | Apolipoprotein C3 | APOC3 |
| Apolipoprotein C-IV | 1 | 0.373 | 0.623 | -1.21 | P55056 | Apolipoprotein C4 | APOC4 |
| Apolipoprotein D | 4 | 0.380 | 0.623 | -1.09 | P05090 | ApoD | APOD |
| Apolipoprotein E | 14 | 0.120 | 0.331 | -1.25 | P02649 | Apo-E | APOE |
| Apolipoprotein F | 2 | 0.904 | 0.919 | -1.02 | Q13790 | LTIP | APOF |
| Apolipoprotein L1 | 5 | 0.310 | 0.574 | -1.14 | O14791 | ApoL-I | APOL1 |
| Apolipoprotein M | 5 | 0.896 | 0.919 | -1.01 | O95445 | Protein G3a | APOM |
| ATP-binding cassette sub-family B member 9 | 1 | 0.088 | 0.273 | 1.48 | Q9NP78 | TAPL | ABCB9 |
| Attractin | 10 | 0.591 | 0.757 | 1.05 | O75882 | Mahogany homolog | ATRN |
| Beta-2-glycoprotein 1 | 11 | 0.007 | 0.053 | -1.24 | P02749 | Beta | APOH |
| Beta-2-microglobulin | 1 | 0.402 | 0.635 | -1.08 | P61769 | Beta-2-microglobulin | B2M |
| Beta-Ala-His dipeptidase | 6 | 0.192 | 0.437 | -1.23 | Q96KN2 | Serum carnosinase | CNDP1 |
| Beta-enolase | 1 | NA | NA | NA | P13929 | Skeletal muscle enolase | ENO3 |
| Biotinidase | 4 | 0.061 | 0.218 | -1.19 | P43251 | EC 3.5.1.12 | BTD |
| C4b-binding protein alpha chain | 10 | 0.478 | 0.697 | -1.07 | P04003 | PRP | C4BPA |
| C4b-binding protein beta chain | 1 | 0.747 | 0.847 | -1.07 | P20851 | C4b-binding protein beta chain | C4BPB |
| Cadherin-1 | 1 | NA | NA | NA | P12830 | CD antigen CD324 | CDH1 |
| Cadherin-5 | 1 | 0.304 | 0.572 | -1.09 | P33151 | CD antigen CD144 | CDH5 |
| Carbonic anhydrase 1 | 3 | 0.194 | 0.437 | -3.67 | P00915 | CA-I | CA1 |
| Carboxypeptidase B2 | 4 | 0.064 | 0.220 | -1.12 | Q96IY4 | TAFI | CPB2 |
| Carboxypeptidase N catalytic chain | 4 | 0.725 | 0.847 | -1.02 | P15169 | SCPN | CPN1 |
| Carboxypeptidase N subunit 2 | 12 | 0.035 | 0.147 | -1.13 | P22792 | Carboxypeptidase N regulatory subunit | CPN2 |
| Cartilage acidic protein 1 | 1 | NA | NA | NA | Q9NQ79 | ASPIC | CRTAC1 |
| Cartilage oligomeric matrix protein | 3 | 0.181 | 0.431 | 1.47 | P49747 | TSP5 | COMP |
| **Caspase-8** | **1** | **0.005** | **0.044** | **1.92** | **Q14790** | **MACH** | **CASP8** |
| CD44 antigen | 1 | 0.574 | 0.753 | 1.13 | P16070 | CD antigen CD44 | CD44 |
| CD5 antigen-like | 3 | 0.094 | 0.286 | 1.23 | O43866 | SP-alpha | CD5L |
| Ceruloplasmin | 26 | 0.434 | 0.673 | -1.04 | P00450 | Ferroxidase | CP |
| Cholesteryl ester transfer protein | 1 | NA | NA | NA | P11597 | Lipid transfer protein I | CETP |
| Cholinesterase | 2 | 0.055 | 0.203 | -1.45 | P06276 | Pseudocholinesterase | BCHE |
| Clathrin heavy chain 1 | 1 | 0.387 | 0.623 | 1.27 | Q00610 | CLH-17 | CLTC |
| Clusterin | 12 | 0.009 | 0.063 | -1.15 | P10909 | Complement cytolysis inhibitor b chain | CLU |
| **Coagulation factor IX** | **6** | **0.003** | **0.035** | **-1.35** | **P00740** | **PTC** | **F9** |
| Coagulation factor V | 11 | 0.640 | 0.791 | 1.03 | P12259 | Proaccelerin. labile factor | F5 |
| Coagulation factor X | 6 | 0.632 | 0.785 | -1.08 | P00742 | Stuart-Prower factor | F10 |
| Coagulation factor XI | 5 | 0.292 | 0.566 | -1.11 | P03951 | PTA | F11 |
| Coagulation factor XII | 9 | 0.143 | 0.366 | -1.17 | P00748 | Beta-factor XIIa part 2 | F12 |
| Coagulation factor XIII A chain | 9 | 0.739 | 0.847 | -1.05 | P00488 | Transglutaminase A chain | F13A1 |
| Coagulation factor XIII B chain | 5 | 0.019 | 0.100 | -1.16 | P05160 | Transglutaminase B chain | F13B |
| Cofilin-1 | 1 | NA | NA | NA | P23528 | Cofilin. non-muscle isoform | CFL1 |
| Collagen type IV alpha-3-binding protein | 1 | 0.035 | 0.147 | 1.33 | Q9Y5P4 | StAR-related lipid transfer protein 11 | COL4A3BP |
| Collectin-11 | 1 | 0.781 | 0.865 | -1.17 | Q9BWP8 | CL-K1 | COLEC11 |
| Complement C1q subcomponent subunit A | 2 | 0.122 | 0.331 | 1.11 | P02745 | Complement C1q subcomponent subunit A | C1QA |
| **Complement C1q subcomponent subunit B** | **4** | **0.005** | **0.044** | **-1.16** | **P02746** | **Complement C1q subcomponent subunit B** | **C1QB** |
| **Complement C1q subcomponent subunit C** | **4** | **0.005** | **0.044** | **-1.16** | **P02747** | **Complement C1q subcomponent subunit C** | **C1QC** |
| Complement C1r subcomponent | 15 | 0.022 | 0.111 | -1.12 | P00736 | Complement component 1 subcomponent r | C1R |
| Complement C1r subcomponent-like protein | 1 | 0.024 | 0.118 | -1.27 | Q9NZP8 | CLSPa | C1RL |
| Complement C1s subcomponent | 15 | 0.033 | 0.144 | -1.09 | P09871 | Complement component 1 subcomponent s | C1S |
| Complement C2 | 13 | 0.051 | 0.193 | -1.10 | P06681 | C3/C5 convertase | C2 |
| Complement C3 | 79 | 0.203 | 0.450 | -1.09 | P01024 | C3adesArg; Complement C3b alpha' chain; Complement C3c alpha' chain fragment 1; Complement C3dg fragment; Complement C3g fragment; Complement C3d fragment; Complement C3f fragment; Complement C3c alpha' chain fragment 2 | C3 |
| Complement C4-A | 4 | 0.352 | 0.623 | 1.13 | P0C0L4 | C3 and PZP-like alpha-2-macroglobulin domain-containing protein 2 | C4A |
| Complement C4-B | 4 | 0.750 | 0.847 | 1.03 | P0C0L5 | C3 and PZP-like alpha-2-macroglobulin domain-containing protein 3 | C4B |
| Complement C5 | 46 | 0.008 | 0.056 | -1.17 | P01031 | C3 and PZP-like alpha-2-macroglobulin domain-containing protein 4 | C5 |
| Complement component C6 | 17 | 0.493 | 0.701 | -1.04 | P13671 | Complement component C6 | C6 |
| Complement component C7 | 18 | 0.573 | 0.753 | -1.05 | P10643 | Complement component C7 | C7 |
| Complement component C8 alpha chain | 10 | 0.472 | 0.694 | -1.04 | P07357 | Complement component 8 subunit alpha | C8A |
| Complement component C8 beta chain | 16 | 0.658 | 0.804 | -1.04 | P07358 | Complement component 8 subunit beta | C8B |
| Complement component C8 gamma chain | 4 | 0.267 | 0.532 | -1.07 | P07360 | Complement component C8 gamma chain | C8G |
| Complement component C9 | 14 | 0.901 | 0.919 | 1.01 | P02748 | Complement component C9 | C9 |
| Complement factor B | 23 | 0.316 | 0.581 | -1.06 | P00751 | Properdin factor B | CFB |
| **Complement factor H** | **40** | **0.000** | **0.018** | **-1.25** | **P08603** | **H factor 1** | **CFH** |
| Complement factor H-related protein 1 | 2 | 0.380 | 0.623 | 1.10 | Q03591 | H36 | CFHR1 |
| Complement factor H-related protein 2 | 3 | 0.185 | 0.435 | -1.24 | P36980 | H factor-like protein 2 | CFHR2 |
| Complement factor H-related protein 5 | 2 | 0.343 | 0.612 | -1.21 | Q9BXR6 | FHR-5 | CFHR5 |
| Complement factor I | 19 | 0.087 | 0.273 | -1.16 | P05156 | C3B/C4B inactivator | CFI |
| Cornifin-B | 1 | NA | NA | NA | P22528 | SPR-IB | SPRR1B |
| Coronin-1A | 1 | NA | NA | NA | P31146 | TACO | CORO1A |
| **Cortactin-binding protein 2** | **1** | **0.003** | **0.039** | **1.76** | **Q8WZ74** | **CortBP2** | **CTTNBP2** |
| Corticosteroid-binding globulin | 7 | 0.026 | 0.124 | -1.16 | P08185 | Transcortin | SERPINA6 |
| C-reactive protein | 4 | 0.761 | 0.847 | 1.11 | P02741 | C-reactive protein | CRP |
| Cyclin-T2 | 1 | 0.852 | 0.914 | 1.01 | O60583 | CycT2 | CCNT2 |
| Cystatin-C | 2 | 0.696 | 0.834 | -1.04 | P01034 | Post-gamma-globulin | CST3 |
| Cytochrome c oxidase subunit 5A mitochondrial | 1 | NA | NA | NA | P20674 | Cytochrome c oxidase polypeptide Va | COX5A |
| Death-associated protein kinase 1 | 1 | 0.897 | 0.919 | -1.01 | P53355 | EC 2.7.11.1 | DAPK1 |
| Desmocollin-1 | 1 | NA | NA | NA | Q08554 | DG2/DG3 | DSC1 |
| Desmoglein-1 | 1 | NA | NA | NA | Q02413 | Pemphigus foliaceus antigen | DSG1 |
| Dopamine beta-hydroxylase | 1 | NA | NA | NA | P09172 | Dopamine beta-monooxygenase | DBH |
| **Dynein heavy chain 12 axonemal** | **1** | **0.002** | **0.025** | **1.61** | **Q6ZR08** | **Dynein heavy chain domain-containing protein 2** | **DNAH12** |
| **E3 ubiquitin-protein ligase TRIM33** | **1** | **0.006** | **0.046** | **1.39** | **Q9UPN9** | **Tripartite motif-containing protein 33** | **TRIM33** |
| EGF-containing fibulin-like extracellular matrix protein 1 | 6 | 0.890 | 0.919 | 1.01 | Q12805 | FIBL-3 | EFEMP1 |
| **Endonuclease 8-like 3** | **1** | **0.004** | **0.041** | **1.32** | **Q8TAT5** | **Nei-like protein 3** | **NEIL3** |
| Extracellular matrix protein 1 | 6 | 0.181 | 0.431 | -1.13 | Q16610 | Secretory component p85 | ECM1 |
| Fermitin family homolog 3 | 4 | 0.759 | 0.847 | -1.14 | Q86UX7 | Unc-112-related protein 2 | FERMT3 |
| Fetuin-B | 4 | 0.015 | 0.089 | -1.26 | Q9UGM5 | Gugu | FETUB |
| Fibrinogen alpha chain | 27 | 0.739 | 0.847 | 1.03 | P02671 | Fibrinogen alpha chain | FGA |
| Fibrinogen beta chain | 23 | 0.561 | 0.751 | 1.06 | P02675 | Fibrinogen beta chain | FGB |
| Fibrinogen gamma chain | 19 | 0.686 | 0.827 | 1.04 | P02679 | Fibrinogen gamma chain | FGG |
| Fibronectin | 62 | 0.710 | 0.841 | -1.06 | P02751 | CIG | FN1 |
| Fibulin-1 | 9 | 0.016 | 0.090 | 1.29 | P23142 | FIBL-1 | FBLN1 |
| Ficolin-3 | 5 | 0.054 | 0.202 | -1.25 | O75636 | Hakata antigen | FCN3 |
| Filaggrin-2 | 1 | NA | NA | NA | Q5D862 | Ifapsoriasin | FLG2 |
| Filamin-A | 16 | 0.557 | 0.750 | -1.54 | P21333 | Non-muscle filamin | FLNA |
| Flavin reductase | 1 | NA | NA | NA | P30043 | FLR | BLVRB |
| Fructose-bisphosphate aldolase B | 1 | NA | NA | NA | P05062 | Liver-type aldolase | ALDOB |
| Galectin-3-binding protein | 4 | 0.142 | 0.365 | -1.27 | Q08380 | Tumor-associated antigen 90K | LGALS3BP |
| Galectin-7 | 1 | NA | NA | NA | P47929 | p53-induced gene 1 protein | LGALS7 |
| Gelsolin | 13 | 0.040 | 0.162 | -1.16 | P06396 | Brevin | GSN |
| Glutathione peroxidase 3 | 3 | 0.011 | 0.075 | -1.18 | P22352 | GSHPx-P | GPX3 |
| Glyceraldehyde-3-phosphate dehydrogenase | 3 | 0.548 | 0.743 | -1.47 | P04406 | EC 2.6.99.- | GAPDH |
| GTPase Era mitochondrial | 1 | NA | NA | NA | O75616 | ERA-like protein 1 | ERAL1 |
| Haptoglobin | 4 | 0.791 | 0.872 | 1.06 | P00738 | Zonulin | HP |
| Haptoglobin-related protein | 1 | NA | NA | NA | P00739 | Haptoglobin-related protein | HPR |
| Hemoglobin subunit alpha | 1 | NA | NA | NA | P69905 | Hemoglobin alpha chain | HBA1; |
| Hemoglobin subunit beta | 3 | 0.270 | 0.533 | -2.85 | P68871 | Hemoglobin beta chain | HBB |
| Hemopexin | 16 | 0.029 | 0.133 | -1.15 | P02790 | Beta-1B-glycoprotein | HPX |
| Heparin cofactor 2 | 16 | 0.242 | 0.499 | -1.10 | P05546 | Serpin D1 | SERPIND1 |
| **Hepatocyte growth factor activator** | **6** | **0.001** | **0.018** | **-1.27** | **Q04756** | **EC 3.4.21.** | **HGFAC** |
| Hepatocyte growth factor-like protein | 5 | 0.835 | 0.899 | 1.02 | P26927 | MSP | MST1 |
| Heterogeneous nuclear ribonucleoprotein C-like 2 | 1 | 0.308 | 0.574 | 1.08 | B2RXH8 | hnRNP C-like-2 | HNRNPCL2 |
| Histidine-rich glycoprotein | 11 | 0.195 | 0.437 | -1.13 | P04196 | HPRG | HRG |
| Hyaluronan-binding protein 2 | 6 | 0.166 | 0.406 | -1.09 | Q14520 | Plasma hyaluronan-binding protein | HABP2 |
| Ig alpha-1 chain C region | 9 | 0.813 | 0.885 | 1.03 | P01876 | Ig alpha-1 chain C region TRO | IGHA1 |
| Ig gamma-1 chain C region | 2 | 0.755 | 0.847 | 1.04 | P01857 | Ig gamma-1 chain C region NIE | IGHG1 |
| Ig gamma-2 chain C region | 1 | 0.728 | 0.847 | 1.06 | P01859 | Ig gamma-2 chain C region ZIE | IGHG2 |
| Ig gamma-3 chain C region | 2 | 0.157 | 0.387 | 1.53 | P01860 | Ig gamma-3 chain C region | IGHG3 |
| Ig gamma-4 chain C region | 2 | 0.192 | 0.437 | -2.29 | P01861 | Ig gamma-4 chain C region | IGHG4 |
| Ig heavy chain V-III region TIL | 1 | 0.471 | 0.694 | 1.21 | P01764 | Ig heavy chain V-III region ZAP | IGHV3-23 |
| Ig heavy chain V-III region WEA | 1 | 0.016 | 0.092 | 1.56 | P01763 | Ig heavy chain V-III region WEA | IGHV3-48 |
| Ig kappa chain C region | 5 | 0.802 | 0.880 | -1.03 | P01834 | Ig kappa chain C region TI | IGKC |
| Ig kappa chain V-I region EU | 1 | NA | NA | NA | P01602 | Ig kappa chain V-I region Kue | IGKV1-5 |
| Ig kappa chain V-III region B6 | 1 | 0.135 | 0.351 | 1.92 | P01619 | Ig kappa chain V-III region WOL | IGKV3-20 |
| Ig kappa chain V-III region SIE | 1 | 0.135 | 0.351 | 1.92 | P01619 | Ig kappa chain V-III region WOL | IGKV3-20 |
| Ig kappa chain V-IV region Len | 1 | 0.328 | 0.593 | -1.26 | P06312 | Ig kappa chain V-IV region STH | IGKV4-1 |
| Ig lambda chain V-IV region Hil | 1 | NA | NA | NA | P01717 | Ig lambda chain V-IV region Hil | IGLV3-25 |
| Ig lambda-2 chain C regions | 4 | 0.375 | 0.623 | 1.11 | P0DOY2 | Ig lambda-2 chain C region | IGLC2 |
| Ig mu chain C region | 10 | 0.112 | 0.320 | 1.33 | P01871 | Ig mu chain C region OU | IGHM |
| IgGFc-binding protein | 8 | 0.026 | 0.124 | 1.21 | Q9Y6R7 | Fcgamma-binding protein antigen | FCGBP |
| Immunoglobulin J chain | 1 | 0.182 | 0.431 | 1.31 | P01591 | Joining chain of multimeric IgA and IgM | JCHAIN |
| Insulin-like growth factor-binding protein 2 | 2 | 0.607 | 0.762 | 1.17 | P18065 | IGFBP-2 | IGFBP2 |
| Insulin-like growth factor-binding protein 3 | 3 | 0.123 | 0.331 | -1.14 | P17936 | IGFBP-3 | IGFBP3 |
| Insulin-like growth factor-binding protein complex acid labile subunit | 11 | 0.033 | 0.144 | -1.36 | P35858 | ALS | IGFALS |
| Inter-alpha-trypsin inhibitor heavy chain H1 | 19 | 0.032 | 0.143 | -1.13 | P19827 | SHAP | ITIH1 |
| Inter-alpha-trypsin inhibitor heavy chain H2 | 23 | 0.448 | 0.681 | -1.06 | P19823 | SHAP | ITIH2 |
| Inter-alpha-trypsin inhibitor heavy chain H3 | 10 | 0.480 | 0.697 | 1.08 | Q06033 | SHAP | ITIH3 |
| Inter-alpha-trypsin inhibitor heavy chain H4 | 29 | 0.965 | 0.965 | 1.00 | Q14624 | PK-120 | ITIH4 |
| Intercellular adhesion molecule 1 | 1 | NA | NA | NA | P05362 | CD antigen CD54 | ICAM1 |
| Intercellular adhesion molecule 2 | 1 | NA | NA | NA | P13598 | CD antigen CD102 | ICAM2 |
| **Kallistatin** | **8** | **0.001** | **0.018** | **-1.35** | **P29622** | **Serpin A4** | **SERPINA4** |
| Keratin type I cuticular Ha1 | 3 | 0.483 | 0.698 | -1.55 | Q15323 | K31 | KRT31 |
| Keratin type I cuticular Ha4 | 1 | NA | NA | NA | O76011 | K34 | KRT34 |
| Keratin type I cytoskeletal 10 | 11 | 0.934 | 0.938 | 1.02 | P13645 | K10 | KRT10 |
| Keratin type I cytoskeletal 13 | 2 | 0.819 | 0.886 | -1.13 | P13646 | K13 | KRT13 |
| Keratin type I cytoskeletal 14 | 2 | NA | NA | NA | P02533 | K14 | KRT14 |
| Keratin type I cytoskeletal 16 | 6 | 0.687 | 0.827 | -1.32 | P08779 | K16 | KRT16 |
| Keratin type I cytoskeletal 17 | 2 | NA | NA | NA | Q04695 | K17 | KRT17 |
| Keratin type I cytoskeletal 9 | 18 | 0.469 | 0.694 | -1.30 | P35527 | K9 | KRT9 |
| Keratin type II cuticular Hb5 | 1 | NA | NA | NA | P78386 | Type-II keratin Kb25 | KRT85 |
| Keratin type II cytoskeletal 1 | 18 | 0.358 | 0.623 | -1.36 | P04264 | Type-II keratin Kb1 | KRT1 |
| Keratin type II cytoskeletal 2 epidermal | 12 | 0.423 | 0.659 | -1.19 | P35908 | Type-II keratin Kb2 | KRT2 |
| Keratin type II cytoskeletal 5 | 4 | 0.209 | 0.458 | -1.18 | P13647 | Type-II keratin Kb5 | KRT5 |
| Keratin type II cytoskeletal 6A | 1 | NA | NA | NA | P02538 | allergen Hom s 5 | KRT6A |
| Keratin type II cytoskeletal 72 | 1 | 0.755 | 0.847 | -1.41 | Q14CN4 | Type-II keratin Kb35 | KRT72 |
| Keratin-associated protein 11-1 | 1 | NA | NA | NA | Q8IUC1 | High sulfur keratin-associated protein 11.1 | KRTAP11-1 |
| Keratin-associated protein 3-1 | 1 | NA | NA | NA | Q9BYR8 | Keratin-associated protein 3.1 | KRTAP3-1 |
| **Kin of IRRE-like protein 3** | **1** | **0.005** | **0.044** | **1.47** | **Q8IZU9** | **Nephrin-like protein 2** | **KIRREL3** |
| Kininogen-1 | 13 | 0.296 | 0.566 | -1.06 | P01042 | Kallidin II; Kininogen-1 light chain; Low molecular weight growth-promoting factor | KNG1 |
| Leucine-rich alpha-2-glycoprotein | 12 | 0.389 | 0.623 | 1.08 | P02750 | LRG | LRG1 |
| Leucine-rich repeat-containing protein 43 | 1 | 0.296 | 0.566 | 1.19 | Q8N309 | Leucine-rich repeat-containing protein 43 | LRRC43 |
| Lipopolysaccharide-binding protein | 6 | 0.891 | 0.919 | 1.02 | P18428 | LBP | LBP |
| L-lactate dehydrogenase A chain | 1 | 0.120 | 0.331 | 1.37 | P00338 | Renal carcinoma antigen NY-REN-59 | LDHA |
| LON peptidase N-terminal domain and RING finger protein 3 | 1 | 0.500 | 0.701 | 1.05 | Q496Y0 | RING finger protein 127 | LONRF3 |
| Long-chain fatty acid transport protein 6 | 1 | 0.044 | 0.173 | -1.16 | Q9Y2P4 | hVLCS-H1 | SLC27A6 |
| **L-selectin** | **2** | **0.000** | **0.018** | **-1.75** | **P14151** | **CD antigen CD62L** | **SELL** |
| Lumican | 7 | 0.595 | 0.758 | -1.04 | P51884 | KSPG lumican | LUM |
| Lysozyme C | 2 | 0.102 | 0.298 | -1.19 | P61626 | 1.4-beta-N-acetylmuramidase C | LYZ |
| Mannan-binding lectin serine protease 1 | 6 | 0.402 | 0.635 | -1.08 | P48740 | Serine protease 5 | MASP1 |
| Mannan-binding lectin serine protease 2 | 2 | 0.385 | 0.623 | -1.15 | O00187 | MASP-2 | MASP2 |
| Mannose-binding protein C | 5 | 0.065 | 0.220 | 2.32 | P11226 | Mannose-binding lectin | MBL2 |
| **Mannosyl-oligosaccharide 12-alpha-mannosidase IC** | **1** | **0.001** | **0.018** | **1.54** | **Q9NR34** | **Alpha-1.2-mannosidase IC** | **MAN1C1** |
| Monocyte differentiation antigen CD14 | 1 | 0.224 | 0.483 | 2.66 | P08571 | CD antigen CD14 | CD14 |
| Multiple inositol polyphosphate phosphatase 1 | 1 | 0.099 | 0.293 | -1.33 | Q9UNW1 | Ins | MINPP1 |
| N-acetylmuramoyl-L-alanine amidase | 10 | 0.074 | 0.246 | -1.13 | Q96PD5 | PGRP-L | PGLYRP2 |
| Neutrophil defensin 1 | 1 | 0.463 | 0.694 | 1.15 | P59665 | HP2 | DEFA1 |
| **Nicotinamide/nicotinic acid mononucleotide adenylyltransferase 3** | **1** | **0.001** | **0.018** | **1.33** | **Q96T66** | **EC 2.7.7.1** | **NMNAT3** |
| Nuclear pore glycoprotein p62 | 1 | 0.010 | 0.065 | 2.09 | P37198 | Nucleoporin Nup62 | NUP62 |
| Pantetheinase | 1 | 0.103 | 0.298 | -1.51 | O95497 | Vanin-1 | VNN1 |
| **Peptidase inhibitor 16** | **1** | **0.001** | **0.020** | **-1.69** | **Q6UXB8** | **PSP94-binding protein** | **PI16** |
| Peptidyl-prolyl cis-trans isomerase A | 1 | NA | NA | NA | P62937 | Rotamase A | PPIA |
| **Phosphatidylcholine-sterol acyltransferase** | **5** | **0.001** | **0.018** | **-1.25** | **P04180** | **Phospholipid-cholesterol acyltransferase** | **LCAT** |
| Phosphatidylinositol-glycan-specific phospholipase D | 11 | 0.487 | 0.700 | 1.08 | P80108 | GPI-specific phospholipase D | GPLD1 |
| Phosphoglycerate kinase 1 | 1 | NA | NA | NA | P00558 | PRP 2 | PGK1 |
| Phospholipid transfer protein | 2 | 0.260 | 0.523 | 1.09 | P55058 | Lipid transfer protein II | PLTP |
| Pigment epithelium-derived factor | 11 | 0.019 | 0.100 | -1.22 | P36955 | Serpin F1 | SERPINF1 |
| Plakophilin-1 | 1 | NA | NA | NA | Q13835 | B6P | PKP1 |
| Plasma kallikrein | 16 | 0.814 | 0.885 | -1.01 | P03952 | PKK | KLKB1 |
| Plasma protease C1 inhibitor | 14 | 0.500 | 0.701 | -1.03 | P05155 | Serpin G1 | SERPING1 |
| Plasma serine protease inhibitor | 5 | 0.007 | 0.054 | -1.32 | P05154 | Serpin A5 | SERPINA5 |
| **Plasminogen** | **22** | **0.001** | **0.018** | **-1.19** | **P00747** | **EC 3.4.21.7** | **PLG** |
| Plastin-2 | 1 | 0.544 | 0.741 | 1.09 | P13796 | LCP-1 | LCP1 |
| Platelet basic protein | 3 | 0.320 | 0.583 | -1.19 | P02775 | NAP-2 | PPBP |
| Platelet factor 4 | 1 | 0.389 | 0.623 | -1.31 | P02776 | Oncostatin-A | PF4 |
| Platelet factor 4 variant | 1 | NA | NA | NA | P10720 | PF4var1 | PF4V1 |
| Platelet glycoprotein Ib alpha chain | 3 | 0.583 | 0.753 | -1.15 | P07359 | CD antigen CD42b | GP1BA |
| Pregnancy zone protein | 5 | 0.122 | 0.331 | -4.49 | P20742 | C3 and PZP-like alpha-2-macroglobulin domain-containing protein 6 | PZP |
| Prenylcysteine oxidase 1 | 1 | 0.381 | 0.623 | 1.57 | Q9UHG3 | Prenylcysteine lyase | PCYOX1 |
| Profilin-1 | 2 | 0.368 | 0.623 | -1.10 | P07737 | Profilin I | PFN1 |
| **Properdin** | **3** | **0.005** | **0.044** | **-1.23** | **P27918** | **Complement factor P** | **CFP** |
| Prostaglandin-H2 D-isomerase | 1 | NA | NA | NA | P41222 | PGDS2 | PTGDS |
| Protein AMBP [Cleaved into: Alpha-1-microglobulin | 9 | 0.024 | 0.118 | -1.13 | P02760 | Uronic-acid-rich protein; Trypstatin | AMBP |
| Protein S100-A9 | 1 | 0.574 | 0.753 | -1.44 | P06702 | S100 calcium-binding protein A9 | S100A9 |
| Protein TANC2 | 1 | 0.013 | 0.081 | 1.54 | Q9HCD6 | Tetratricopeptide repeat. ankyrin repeat and coiled-coil domain-containing protein 2 | TANC2 |
| Protein Z-dependent protease inhibitor | 3 | 0.153 | 0.381 | -1.22 | Q9UK55 | Serpin A10 | SERPINA10 |
| **Protein-glutamine gamma-glutamyltransferase 6** | **1** | **0.005** | **0.044** | **1.41** | **O95932** | **TGase-6** | **TGM6** |
| Prothrombin | 14 | 0.037 | 0.152 | -1.21 | P00734 | Coagulation factor II | F2 |
| **Protocadherin-15** | **1** | **0.006** | **0.046** | **-1.20** | **Q96QU1** | **Protocadherin-15** | **PCDH15** |
| **Putative heat shock 70 kDa protein 7** | **1** | **0.001** | **0.018** | **1.77** | **P48741** | **Heat shock 70 kDa protein B** | **HSPA7** |
| Putative hydroxypyruvate isomerase | 1 | 0.058 | 0.210 | 1.24 | Q5T013 | Endothelial cell apoptosis protein E-CE1 | HYI |
| Putative keratin-87 protein | 1 | NA | NA | NA | A6NCN2 | Keratin-121 pseudogene | KRT87P |
| **Ras-related GTP-binding protein B** | **1** | **0.003** | **0.039** | **1.44** | **Q5VZM2** | **RagB** | **RRAGB** |
| Retinol-binding protein 4 | 8 | 0.028 | 0.132 | -1.26 | P02753 | RBP | RBP4 |
| Rho GDP-dissociation inhibitor 2 | 1 | 0.750 | 0.847 | 1.24 | P52566 | Rho-GDI beta | ARHGDIB |
| Secreted phosphoprotein 24 | 1 | NA | NA | NA | Q13103 | Secreted phosphoprotein 2 | SPP2 |
| Selenoprotein P | 5 | 0.232 | 0.495 | -1.10 | P49908 | SeP | SELENOP |
| Serglycin | 1 | NA | NA | NA | P10124 | Secretory granule proteoglycan core protein | SRGN |
| **Serine palmitoyltransferase 2** | **1** | **0.002** | **0.025** | **1.89** | **O15270** | **SPT 2** | **SPTLC2** |
| Serotransferrin | 18 | 0.081 | 0.267 | 1.15 | P02787 | Siderophilin | TF |
| Serpin B3 | 1 | NA | NA | NA | P29508 | SCCA-1 | SERPINB3 |
| Serum albumin | 29 | 0.544 | 0.741 | -1.06 | P02768 | Serum albumin | ALB |
| Serum amyloid A-1 protein | 3 | 0.240 | 0.499 | 1.66 | P0DJI8 | Amyloid fibril protein AA; Serum amyloid protein A | SAA1 |
| Serum amyloid A-4 protein | 6 | 0.386 | 0.623 | 1.05 | P35542 | C-SAA | SAA4 |
| Serum amyloid P-component | 6 | 0.062 | 0.218 | -1.17 | P02743 | 9.5S alpha-1-glycoprotein | APCS |
| Serum paraoxonase/arylesterase 1 | 9 | 0.148 | 0.372 | -1.15 | P27169 | Serum aryldialkylphosphatase 1 | PON1 |
| Serum paraoxonase/lactonase 3 | 2 | 0.285 | 0.558 | -1.11 | Q15166 | EC 3.1.8.1 | PON3 |
| Sex hormone-binding globulin | 4 | 0.645 | 0.792 | 1.13 | P04278 | Testosterone-estrogen-binding globulin | SHBG |
| Sialic acid-binding Ig-like lectin 16 | 1 | 0.241 | 0.499 | 1.13 | A6NMB1 | Siglec-P16 | SIGLEC16 |
| Signal transducer and activator of transcription 1-alpha/beta | 1 | 0.500 | 0.701 | 1.05 | P42224 | Transcription factor ISGF-3 components p91/p84 | STAT1 |
| Sulfhydryl oxidase 1 | 2 | 0.870 | 0.919 | -1.02 | O00391 | Quiescin Q6 | QSOX1 |
| **SWI/SNF complex subunit SMARCC1** | **1** | **0.001** | **0.018** | **2.49** | **Q92922** | **SWI/SNF-related matrix-associated actin-dependent regulator of chromatin subfamily C member 1** | **SMARCC1** |
| Talin-1 | 11 | 0.084 | 0.270 | -2.40 | Q9Y490 | Talin-1 | TLN1 |
| Tetranectin | 7 | 0.012 | 0.079 | -1.14 | P05452 | Plasminogen kringle 4-binding protein | CLEC3B |
| **Tetratricopeptide repeat protein 39A** | **1** | **0.001** | **0.018** | **2.04** | **Q5SRH9** | **DEME-6** | **TTC39A** |
| THAP domain-containing protein 4 | 1 | 0.875 | 0.919 | 1.04 | Q8WY91 | THAP domain-containing protein 4 | THAP4 |
| Thrombospondin-1 | 8 | 0.242 | 0.499 | -1.22 | P07996 | Thrombospondin-1 | THBS1 |
| **Thyroxine-binding globulin** | **11** | **0.001** | **0.018** | **-1.29** | **P05543** | **T4-binding globulin** | **SERPINA7** |
| Titin | 2 | 0.521 | 0.722 | 1.06 | Q8WZ42 | Rhabdomyosarcoma antigen MU-RMS-40.14 | TTN |
| **Transcription elongation factor SPT6** | **1** | **0.000** | **0.018** | **1.83** | **Q7KZ85** | **Tat-CT2 protein** | **SUPT6H** |
| Transforming growth factor-beta-induced protein ig-h3 | 1 | 0.709 | 0.841 | 1.09 | Q15582 | RGD-CAP | TGFBI |
| Transgelin-2 | 1 | 0.526 | 0.725 | -1.29 | P37802 | SM22-alpha homolog | TAGLN2 |
| Transthyretin | 6 | 0.132 | 0.351 | -1.17 | P02766 | TBPA | TTR |
| Tropomyosin alpha-4 chain | 2 | 0.259 | 0.523 | -1.51 | P67936 | Tropomyosin-4 | TPM4 |
| Tubulin alpha-1B chain | 2 | 0.907 | 0.919 | -1.11 | P68363 | Tubulin alpha-ubiquitous chain | TUBA1B |
| Vasorin | 1 | NA | NA | NA | Q6EMK4 | Protein slit-like 2 | VASN |
| Vinculin | 5 | 0.717 | 0.845 | 1.12 | P18206 | MV | VCL |
| **Vitamin D-binding protein** | **24** | **0.001** | **0.018** | **-1.22** | **P02774** | **DBP-maf** | **GC** |
| Vitamin K-dependent protein C | 2 | 0.046 | 0.181 | -1.32 | P04070 | Blood coagulation factor XIV | PROC |
| Vitamin K-dependent protein S | 8 | 0.092 | 0.284 | -1.23 | P07225 | Vitamin K-dependent protein S | PROS1 |
| Vitamin K-dependent protein Z | 1 | NA | NA | NA | P22891 | Vitamin K-dependent protein Z | PROZ |
| Vitronectin | 8 | 0.872 | 0.919 | 1.01 | P04004 | V75 | VTN |
| von Willebrand factor | 19 | 0.463 | 0.694 | -1.30 | P04275 | von Willebrand antigen II | VWF |
| **Zinc finger protein 30 homolog** | **1** | **0.001** | **0.018** | **1.75** | **Q9Y2G7** | **Zinc finger protein 745** | **ZFP30** |
| Zinc-alpha-2-glycoprotein | 12 | 0.019 | 0.100 | -1.17 | P25311 | Zn-alpha-2-glycoprotein | AZGP1 |
| Zyxin | 1 | NA | NA | NA | Q15942 | Zyxin-2 | ZYX |
