# Supplementary material for: Circulating proteomic patterns in AF related left atrial remodeling indicate involvement of coagulation and complement cascade
Source: PLoS One. 2018 Nov 29;13(11):e0198461. doi: 10.1371/journal.pone.0198461 (PMC6264811; doi:10.1371/journal.pone.0198461)
Supplement: S2 Table — P-value–derived from T-Test, p-value (FDR)–T-Test data with Benjamini Hochberg false discovery rate, FC–fold change in protein levels. (DOC) [file pone.0198461.s002.doc]

**Supplementary data 2**

**Table**: Plasma proteins found differentially abundant when comparing patients with LAD>44mm compared to patients with LAD<44mm. P-value – derived from T-Test, p-value (FDR) – T-Test data with Benjamini Hochberg false discovery rate, FC – fold change in protein levels.

| **Protein names** | **UP** | **P-Value** | **P-value (FDR)** | **FC** | **UniProt Entry** | **alternative protein name** | **gene symbol** |
| --- | --- | --- | --- | --- | --- | --- | --- |
| Prothrombin | 14 | 0.0016 | 0.1550 | -1.33 | P00734 | Coagulation factor II | F2 |
| Dynein heavy chain 12 axonemal | 1 | 0.0017 | 0.1550 | 1.63 | Q6ZR08 | Dynein heavy chain domain-containing protein 2 | DNAH12 |
| Vitamin K-dependent protein C | 2 | 0.0020 | 0.1550 | -1.52 | P04070 | Blood coagulation factor XIV | PROC |
| L-selectin | 2 | 0.0051 | 0.3009 | -1.57 | P14151 | CD antigen CD62L | SELL |
| Ficolin-3 | 5 | 0.0090 | 0.3170 | -1.36 | O75636 | Hakata antigen | FCN3 |
| Coagulation factor XII | 9 | 0.0106 | 0.3170 | -1.31 | P00748 | Beta-factor XIIa part 2 | F12 |
| Fibulin-1 | 9 | 0.0111 | 0.3170 | 1.31 | P23142 | FIBL-1 | FBLN1 |
| Collectin-11 | 1 | 0.0135 | 0.3170 | 4.07 | Q9BWP8 | CL-K1 | COLEC11 |
| Alpha-1-acid glycoprotein 1 | 6 | 0.0144 | 0.3170 | 1.24 | P02763 | OMD 1 | ORM1 |
| Protein Z-dependent protease inhibitor | 3 | 0.0160 | 0.3170 | -1.39 | Q9UK55 | Serpin A10 | SERPINA10 |
| Vitamin K-dependent protein S | 8 | 0.0168 | 0.3170 | -1.35 | P07225 | Vitamin K-dependent protein S | PROS1 |
| Coagulation factor IX | 6 | 0.0170 | 0.3170 | -1.28 | P00740 | PTC | F9 |
| Ig kappa chain V-IV region Len | 1 | 0.0189 | 0.3170 | -1.73 | P06312 | Ig kappa chain V-IV region STH | IGKV4-1 |
| Apolipoprotein | 10 | 0.0205 | 0.3170 | -2.64 | P08519 | EC 3.4.21.- | LPA |
| Serum amyloid A-1 protein | 3 | 0.0209 | 0.3170 | 2.70 | P0DJI8 | Amyloid fibril protein AA; Serum amyloid protein A | SAA1 |
| E3 ubiquitin-protein ligase TRIM33 | 1 | 0.0215 | 0.3170 | 1.32 | Q9UPN9 | Tripartite motif-containing protein 33 | TRIM33 |
| SWI/SNF complex subunit SMARCC1 | 1 | 0.0249 | 0.3460 | 1.93 | Q92922 | SWI/SNF-related matrix-associated actin-dependent regulator of chromatin subfamily C member 1 | SMARCC1 |
| Tetratricopeptide repeat protein 39A | 1 | 0.0278 | 0.3643 | 1.66 | Q5SRH9 | DEME-6 | TTC39A |
| Cortactin-binding protein 2 | 1 | 0.0311 | 0.3812 | 1.54 | Q8WZ74 | CortBP2 | CTTNBP2 |
| Nicotinamide/nicotinic acid mononucleotide adenylyltransferase 3 | 1 | 0.0323 | 0.3812 | 1.21 | Q96T66 | EC 2.7.7.1 | NMNAT3 |
| Attractin | 10 | 0.0350 | 0.3921 | 1.19 | O75882 | Mahogany homolog | ATRN |
| Inter-alpha-trypsin inhibitor heavy chain H4 | 29 | 0.0365 | 0.3921 | 1.10 | Q14624 | PK-120 | ITIH4 |
| Ig lambda-2 chain C regions | 4 | 0.0390 | 0.3997 | -1.28 | P0DOY2 | Ig lambda-2 chain C region | IGLC2 |
| Protein-glutamine gamma-glutamyltransferase 6 | 1 | 0.0460 | 0.4521 | 1.29 | O95932 | TGase-6 | TGM6 |
| Serine palmitoyltransferase 2 | 1 | 0.0491 | 0.4521 | 1.53 | O15270 | SPT 2 | SPTLC2 |
| Ig heavy chain V-III region TIL | 1 | 0.0498 | 0.4521 | -1.65 | P01764 | Ig heavy chain V-III region ZAP | IGHV3-23 |
