# Supplementary material for: Circulating proteomic patterns in AF related left atrial remodeling indicate involvement of coagulation and complement cascade
Source: PLoS One. 2018 Nov 29;13(11):e0198461. doi: 10.1371/journal.pone.0198461 (PMC6264811; doi:10.1371/journal.pone.0198461)
Supplement: S3 Table — (DOC) [file pone.0198461.s003.doc]

**Supplementary data 3**

**Table:** Peptide data of the proteins significantly different between patient subgroups with and without LVA, UP/RP = unique/ razor peptides, Modifications - CM = Carbamidomethyl, O=Oxidation

| **SP ID** | **Entry name** | **Description** | **UP/RP** | **Peptide** | **Modifications** | **Max Refiner Ionscore** |
| --- | --- | --- | --- | --- | --- | --- |
| P02746 | C1QB | Complement C1q subcomponent subunit B | 4 | VPGLYYFTYHASSR |  | 50.39 |
| P02746 | C1QB | Complement C1q subcomponent subunit B | 4 | FDHVITNMNNNYEPR |  | 82.15 |
| P02746 | C1QB | Complement C1q subcomponent subunit B | 4 | GNLCVNLMR | CM [C4] | 63.60 |
| P02746 | C1QB | Complement C1q subcomponent subunit B | 4 | VVTFCDYAYNTFQVTTGGMVLK | CM [C5] | 133.34 |
| P02747 | C1QC | Complement C1q subcomponent subunit C | 4 | QTHQPPAPNSLIR |  | 42.62 |
| P02747 | C1QC | Complement C1q subcomponent subunit C | 4 | FQSVFTVTR |  | 61.29 |
| P02747 | C1QC | Complement C1q subcomponent subunit C | 4 | VVTFCGHTSK | CM [C5] | 40.63 |
| P02747 | C1QC | Complement C1q subcomponent subunit C | 4 | TNQVNSGGVLLR |  | 67.35 |
| Q14790 | CASP8 | Caspase-8 | 1 | AQISAYR |  | 29.01 |
| P08603 | CFAH | Complement factor H | 40 | AGEQVTYTCATYYK | CM [C9] | 93.43 |
| P08603 | CFAH | Complement factor H | 40 | AQTTVTCMENGWSPTPR | CM [C7] : CM [C7] O [M8] | 101.34 |
| P08603 | CFAH | Complement factor H | 40 | AVYTCNEGYQLLGEINYR | CM [C5] | 116.21 |
| P08603 | CFAH | Complement factor H | 40 | CFEGFGIDGPAIAK | CM [C1] | 88.77 |
| P08603 | CFAH | Complement factor H | 40 | CLPVTAPENGK | CM [C1] | 54.41 |
| P08603 | CFAH | Complement factor H | 40 | CNMGYEYSER | CM [C1] : CM [C1] O [M3] | 56.00 |
| P08603 | CFAH | Complement factor H | 40 | CTSTGWIPAPR | CM [C1] | 70.16 |
| P08603 | CFAH | Complement factor H | 40 | CVEISCK | CM [C1 C6] | 35.40 |
| P08603 | CFAH | Complement factor H | 40 | DGWSAQPTCIK | CM [C9] | 53.20 |
| P08603 | CFAH | Complement factor H | 40 | DTSCVNPPTVQNAYIVSR | CM [C4] | 90.07 |
| P08603 | CFAH | Complement factor H | 40 | ECDTDGWTNDIPICEVVK | CM [C2 C14] | 101.64 |
| P08603 | CFAH | Complement factor H | 40 | EEYGHSEVVEYYCNPR | CM [C13] | 40.91 |
| P08603 | CFAH | Complement factor H | 40 | EFDHNSNIR |  | 59.43 |
| P08603 | CFAH | Complement factor H | 40 | EGWIHTVCINGR | CM [C8] | 58.20 |
| P08603 | CFAH | Complement factor H | 40 | EQVQSCGPPPELLNGNVK | CM [C6] | 95.88 |
| P08603 | CFAH | Complement factor H | 40 | GDAVCTESGWRPLPSCEEK | CM [C5 C16] | 54.64 |
| P08603 | CFAH | Complement factor H | 40 | IDVHLVPDR |  | 42.21 |
| P08603 | CFAH | Complement factor H | 40 | IEGDEEMHCSDDGFWSK | CM [C9] | 84.74 |
| P08603 | CFAH | Complement factor H | 40 | LGYVTADGETSGSITCGK | CM [C16] | 118.59 |
| P08603 | CFAH | Complement factor H | 40 | LSYTCEGGFR | CM [C5] | 75.11 |
| P08603 | CFAH | Complement factor H | 40 | NDFTWFK |  | 42.30 |
| P08603 | CFAH | Complement factor H | 40 | NTEILTGSWSDQTYPEGTQAIYK |  | 101.87 |
| P08603 | CFAH | Complement factor H | 40 | SCDIPVFMNAR | CM [C2] | 61.46 |
| P08603 | CFAH | Complement factor H | 40 | SCDNPYIPNGDYSPLR | CM [C2] | 58.63 |
| P08603 | CFAH | Complement factor H | 40 | SIDVACHPGYALPK | CM [C6] | 70.41 |
| P08603 | CFAH | Complement factor H | 40 | SITCIHGVWTQLPQCVAIDK | CM [C4 C15] | 70.74 |
| P08603 | CFAH | Complement factor H | 40 | SLGNVIMVCR | CM [C9] O [M7] | 41.85 |
| P08603 | CFAH | Complement factor H | 40 | SPDVINGSPISQK |  | 57.77 |
| P08603 | CFAH | Complement factor H | 40 | SPPEISHGVVAHMSDSYQYGEEVTYK |  | 71.53 |
| P08603 | CFAH | Complement factor H | 40 | SSNLIILEEHLK |  | 51.68 |
| P08603 | CFAH | Complement factor H | 40 | SSQESYAHGTK |  | 46.40 |
| P08603 | CFAH | Complement factor H | 40 | TDCLSLPSFENAIPMGEK | CM [C3] | 63.98 |
| P08603 | CFAH | Complement factor H | 40 | TGDEITYQCR | CM [C9] | 60.41 |
| P08603 | CFAH | Complement factor H | 40 | TGESVEFVCK | CM [C9] | 70.63 |
| P08603 | CFAH | Complement factor H | 40 | VGEVLK |  | 41.93 |
| P08603 | CFAH | Complement factor H | 40 | VSVLCQENYLIQEGEEITCK | CM [C5 C19] | 122.35 |
| P08603 | CFAH | Complement factor H | 40 | WQSIPLCVEK | CM [C7] | 29.30 |
| P08603 | CFAH | Complement factor H | 40 | WSSPPQCEGLPCK | CM [C7 C12] | 87.74 |
| P08604 | CFAH | Complement factor H | 40 | SSIDIENGFISESQYTYALK |  | 72.56 |
| P08605 | CFAH | Complement factor H | 40 | ISEENETTCYMGK |  | 62.36 |
| Q8WZ74 | CTTB2 | Cortactin-binding protein 2 | 1 | SLENDLSLTLNLDQR |  | 46.01 |
| Q6ZR08 | DYH12 | Dynein heavy chain 12. axonemal | 1 | IIILR |  | 39.56 |
| P00740 | FA9 | Coagulation factor IX | 6 | DSCQGDSGGPHVTEVEGTSFLTGIISWGEECAMK | CM [C3 C31] | 48.34 |
| P00740 | FA9 | Coagulation factor IX | 6 | FGSGYVSGWGR |  | 83.38 |
| P00740 | FA9 | Coagulation factor IX | 6 | VVCSCTEGYR | CM [C3 C5] | 60.73 |
| P00740 | FA9 | Coagulation factor IX | 6 | NCELDVTCNIK | CM [C2 C8] | 72.21 |
| P00740 | FA9 | Coagulation factor IX | 6 | SCEPAVPFPCGR | CM [C2 C10] | 64.66 |
| P00740 | FA9 | Coagulation factor IX | 6 | VDAFCGGSIVNEK | CM [C5] | 96.26 |
| Q04756 | HGFA | Hepatocyte growth factor activator | 6 | EALVPLVADHK |  | 39.87 |
| Q04756 | HGFA | Hepatocyte growth factor activator | 6 | TTDVTQTFGIEK |  | 41.36 |
| Q04756 | HGFA | Hepatocyte growth factor activator | 6 | VANYVDWINDR |  | 51.96 |
| Q04756 | HGFA | Hepatocyte growth factor activator | 6 | SDACQGDSGGPLACEK | CM [C4 C14] | 76.11 |
| Q04756 | HGFA | Hepatocyte growth factor activator | 6 | VQLSPDLLATLPEPASPGR |  | 114.83 |
| Q04756 | HGFA | Hepatocyte growth factor activator | 6 | CSSPEVYGADISPNMLCAGYFDCK | CM [C1 C17 C23] | 91.00 |
| P48741 | HSP77 | Putative heat shock 70 kDa protein 7 | 1 | DAGAIAGLK |  | 33.47 |
| P29622 | KAIN | Kallistatin | 8 | VGSALFLSHNLK |  | 33.97 |
| P29622 | KAIN | Kallistatin | 8 | WNNLLR |  | 32.13 |
| P29622 | KAIN | Kallistatin | 8 | FFSAQTNR |  | 43.77 |
| P29622 | KAIN | Kallistatin | 8 | WADLSGITK |  | 48.60 |
| P29622 | KAIN | Kallistatin | 8 | LGFTDLFSK |  | 59.59 |
| P29622 | KAIN | Kallistatin | 8 | IAPANADFAFR |  | 100.39 |
| P29622 | KAIN | Kallistatin | 8 | FYYLIASETPGK |  | 95.86 |
| P29622 | KAIN | Kallistatin | 8 | FSISGSYVLDQILPR |  | 93.86 |
| Q8IZU9 | KIRR3 | Kin of IRRE-like protein 3 | 1 | VEIVHK |  | 33.38 |
| P04180 | LCAT | Phosphatidylcholine-sterol acyltransferase | 5 | LDKPDVVNWMCYR | CM [C11] | 36.42 |
| P04180 | LCAT | Phosphatidylcholine-sterol acyltransferase | 5 | TYIYDHGFPYTDPVGVLYEDGDDTVATR |  | 78.81 |
| P04180 | LCAT | Phosphatidylcholine-sterol acyltransferase | 5 | STELCGLWQGR | CM [C5] | 86.21 |
| P04180 | LCAT | Phosphatidylcholine-sterol acyltransferase | 5 | SSGLVSNAPGVQIR |  | 90.27 |
| P04180 | LCAT | Phosphatidylcholine-sterol acyltransferase | 5 | ITTTSPWMFPSR |  | 54.82 |
| P14151 | LYAM1 | L-selectin | 2 | AEIEYLEK |  | 35.51 |
| P14151 | LYAM1 | L-selectin | 2 | TICESSGIWSNPSPICQK | CM [C3 C16] | 81.54 |
| Q9NR34 | MA1C1 | Mannosyl-oligosaccharide 12-alpha-mannosidase IC | 1 | LLPAFNTPTGIPK |  | 37.23 |
| Q8TAT5 | NEIL3 | Endonuclease 8-like 3 | 1 | TTNDITQPSSK |  | 35.68 |
| Q96T66 | NMNA3 | Nicotinamide/nicotinic acid mononucleotide adenylyltransferase 3 | 1 | LLCGADVLK | CM [C3] | 31.53 |
| Q96QU1 | PCD15 | Protocadherin-15 | 1 | VVVESIGAR |  | 35.85 |
| Q6UXB8 | PI16 | Peptidase inhibitor 16 | 1 | WDEELAAFAK |  | 64.45 |
| P00747 | PLMN | Plasminogen | 22 | APWCHTTNSQVR | CM [C4] | 37.27 |
| P00747 | PLMN | Plasminogen | 22 | CQSWSSMTPHR | CM [C1] | 46.36 |
| P00747 | PLMN | Plasminogen | 22 | CTTPPPSSGPTYQCLK | CM [C1 C14] | 89.40 |
| P00747 | PLMN | Plasminogen | 22 | EAQLPVIENK |  | 58.03 |
| P00747 | PLMN | Plasminogen | 22 | FSPATHPSEGLEENYCR | CM [C16] | 85.55 |
| P00747 | PLMN | Plasminogen | 22 | FVTWIEGVMR |  | 63.26 |
| P00747 | PLMN | Plasminogen | 22 | HSIFTPETNPR |  | 55.43 |
| P00747 | PLMN | Plasminogen | 22 | LFLEPTR |  | 53.53 |
| P00747 | PLMN | Plasminogen | 22 | LSSPAVITDK |  | 52.59 |
| P00747 | PLMN | Plasminogen | 22 | LYDYCDVPQCAAPSFDCGKPQVEPK | CM [C5 C10 C17] | 73.23 |
| P00747 | PLMN | Plasminogen | 22 | NLDENYCR | CM [C7] | 36.08 |
| P00747 | PLMN | Plasminogen | 22 | NPDNDPQGPWCYTTDPEK | CM [C11] | 91.84 |
| P00747 | PLMN | Plasminogen | 22 | QLGAGSIEECAAK | CM [C10] | 85.32 |
| P00747 | PLMN | Plasminogen | 22 | TPENFPCK | CM [C7] | 53.27 |
| P00747 | PLMN | Plasminogen | 22 | TPENYPNAGLTMNYCR | CM [C15] : CM [C15] O [M12] | 109.62 |
| P00747 | PLMN | Plasminogen | 22 | VQSTELCAGHLAGGTDSCQGDSGGPLVCFEK | CM [C7 C18 C28] | 87.10 |
| P00747 | PLMN | Plasminogen | 22 | WELCDIPR | CM [C4] | 38.92 |
| P00747 | PLMN | Plasminogen | 22 | YDYCDILECEEECMHCSGENYDGK | CM [C4 C9 C13 C16] | 71.00 |
| P00748 | PLMN | Plasminogen | 22 | ATTVTGTPCQDWAAQEPHR | CM [C9] | 79.43 |
| P00749 | PLMN | Plasminogen | 22 | CEEDEEFTCR | CM [C1 C9] | 67.58 |
| P00750 | PLMN | Plasminogen | 22 | NPDGDVGGPWCYTTNPR | CM [C11] | 89.63 |
| P00751 | PLMN | Plasminogen | 22 | TECFITGWGETQGTFGAGLLK | CM [C3] | 98.36 |
| P27918 | PROP | Properdin | 3 | LCTPLLPK | CM [C2] | 33.29 |
| P27918 | PROP | Properdin | 3 | SISCQEIPGQQSR | CM [C4] | 60.56 |
| P27918 | PROP | Properdin | 3 | TCNHPVPQHGGPFCAGDATR | CM [C2 C14] | 35.76 |
| Q5VZM2 | RRAGB | Ras-related GTP-binding protein B | 1 | IHSLQINSSLSTYSLVDSVG NTK |  | 32.13 |
| Q92922 | SMRC1 | SWI/SNF complex subunit SMARCC1 | 1 | LNPQEYLTSTACR | CM [C12] | 33.81 |
| Q7KZ85 | SPT6H | Transcription elongation factor SPT6 | 1 | FLLNK |  | 34.55 |
| O15270 | SPTC2 | Serine palmitoyltransferase 2 | 1 | ECVQQLAENTR | CM [C2] | 38.44 |
| O95932 | TGM3L | Protein-glutamine gamma-glutamyltransferase 6 | 1 | ASVQFDITPSK |  | 39.58 |
| P05543 | THBG | Thyroxine-binding globulin | 11 | MGIQHAYSENADFSGLTEDNGLK |  | 100.57 |
| P05543 | THBG | Thyroxine-binding globulin | 11 | AVLHIGEK |  | 38.88 |
| P05543 | THBG | Thyroxine-binding globulin | 11 | FTVETPDK |  | 43.43 |
| P05543 | THBG | Thyroxine-binding globulin | 11 | GWVDLFVPK |  | 58.45 |
| P05543 | THBG | Thyroxine-binding globulin | 11 | NALALFVLPK |  | 59.15 |
| P05543 | THBG | Thyroxine-binding globulin | 11 | SFMLLILER |  | 61.13 |
| P05543 | THBG | Thyroxine-binding globulin | 11 | TEDSSSFLIDK |  | 54.49 |
| P05543 | THBG | Thyroxine-binding globulin | 11 | AQWANPFDPSK |  | 58.08 |
| P05543 | THBG | Thyroxine-binding globulin | 11 | EGQMESVEAAMSSK | O [M4] | 94.53 |
| P05543 | THBG | Thyroxine-binding globulin | 11 | MSSINADFAFNLYR |  | 52.65 |
| P05544 | THBG | Thyroxine-binding globulin | 11 | FSISATYDLGATLLK |  | 65.41 |
| Q9UPN9 | TRI33 | E3 ubiquitin-protein ligase TRIM33 | 1 | DTSEAPSSSDEK |  | 32.17 |
| Q5SRH9 | TT39A | Tetratricopeptide repeat protein 39A | 1 | IEVIK |  | 30.42 |
| P02774 | VTDB | Vitamin D-binding protein | 24 | THLPEVFLSK |  | 47.09 |
| P02774 | VTDB | Vitamin D-binding protein | 24 | SCESNSPFPVHPGTAECCTK | CM [C2 C17 C18] | 81.43 |
| P02774 | VTDB | Vitamin D-binding protein | 24 | VPTADLEDVLPLAEDITNILSK |  | 96.68 |
| P02774 | VTDB | Vitamin D-binding protein | 24 | EVVSLTEACCAEGADPDCYDTR | CM [C9 C10 C18] | 140.35 |
| P02774 | VTDB | Vitamin D-binding protein | 24 | HQPQEFPTYVEPTNDEICEAFR | CM [C18] | 68.16 |
| P02774 | VTDB | Vitamin D-binding protein | 24 | SDFASNCCSINSPPLYCDSEIDAELK | CM [C7 C8 C17] | 98.72 |
| P02774 | VTDB | Vitamin D-binding protein | 24 | EYANQFMWEYSTNYGQAPLSLLVSYTK |  | 75.10 |
| P02774 | VTDB | Vitamin D-binding protein | 24 | TAMDVFVCTYFMPAAQLPELPDVELPTNK | CM [C8] : CM [C8] O [M3] | 87.12 |
| P02774 | VTDB | Vitamin D-binding protein | 24 | LPDATPK |  | 35.06 |
| P02774 | VTDB | Vitamin D-binding protein | 24 | VLEPTLK |  | 38.35 |
| P02774 | VTDB | Vitamin D-binding protein | 24 | YTFELSR |  | 38.39 |
| P02774 | VTDB | Vitamin D-binding protein | 24 | ELSSFIDK |  | 45.45 |
| P02774 | VTDB | Vitamin D-binding protein | 24 | LCDNLSTK | CM [C2] | 45.86 |
| P02774 | VTDB | Vitamin D-binding protein | 24 | ELPEHTVK |  | 33.57 |
| P02774 | VTDB | Vitamin D-binding protein | 24 | DVCDPGNTK | CM [C3] | 46.96 |
| P02774 | VTDB | Vitamin D-binding protein | 24 | FEDCCQEK | CM [C4 C5] | 48.58 |
| P02774 | VTDB | Vitamin D-binding protein | 24 | HLSLLTTLSNR |  | 62.24 |
| P02774 | VTDB | Vitamin D-binding protein | 24 | VCSQYAAYGEK | CM [C2] | 61.64 |
| P02774 | VTDB | Vitamin D-binding protein | 24 | CCESASEDCMAK | CM [C1 C2 C9] | 69.77 |
| P02774 | VTDB | Vitamin D-binding protein | 24 | EDFTSLSLVLYSR |  | 77.12 |
| P02774 | VTDB | Vitamin D-binding protein | 24 | FPSGTFEQVSQLVK |  | 85.90 |
| P02774 | VTDB | Vitamin D-binding protein | 24 | GQELCADYSENTFTEYK | CM [C5] | 123.80 |
| P02774 | VTDB | Vitamin D-binding protein | 24 | SLGECCDVEDSTTCFNAK | CM [C5 C6 C14] | 114.28 |
| P02774 | VTDB | Vitamin D-binding protein | 24 | SYLSMVGSCCTSASPTVCFLK | CM [C9 C10 C18] | 120.38 |
| Q9Y2G7 | ZFP30 | Zinc finger protein 30 homolog | 1 | LNIAEK |  | 35.51 |
